# Medico-legal reasoning in disability assessment: A focus group and validation study

**DOI:** 10.1186/1471-2458-8-335

**Published:** 2008-09-25

**Authors:** WEL de Boer, P Donceel, S Brage, M Rus, JHBM Willems

**Affiliations:** 1 Department of Quality of Life, TNO, Hoofddorp, the Netherlands; 2 Section of Occupational, Environmental and Insurance Medicine, Department of Public Health, Katholieke Universiteit Leuven, Leuven, Belgium; 3 Department of General Practice and Community Medicine Faculty of Medicine, University of Oslo, Oslo, Norway; 4 Department of assessment, Pension and invalidity insurance of Slovenia, Ljubljana, Slovenia; 5 Coronel Institute, Amsterdam University, Amsterdam, the Netherlands

## Abstract

**Background:**

Decisions on disability pensions are based, among others, on medical reports. The way these medical assessments are performed is largely unclear. The aim of the study was to determine which grounds are used by social insurance physicians (SIPs) in these assessments and to determine if the identification of these grounds can help improve the quality of assessments in social insurance practice. The article describes a focus group study and a questionnaire study with SIPs in four different countries.

**Method:**

Using focus group discussions of SIPs discussing the same case in Belgium, the Netherlands, Norway and Slovenia (N = 29) we determined the arguments and underlying grounds as used by the SIP's. We used a questionnaire study among other SIPs (N = 60) in the same countries to establish a first validation of these grounds.

**Results:**

Grounds in the focus groups were comparable between the countries studied. The grounds were also recognized by SIPs who had not participated in the focus groups. SIPs agreed most on grounds with regard to the claimant's health condition, and about the claimant's duty to explore rehabilitation and work resumption, but less on accepting permanent incapacity when all options for treatment were exhausted.

**Conclusion:**

Grounds that SIPs use refer to a limited group of key elements of disability evaluation. SIPs interpret disability in social insurance according to the handicapped role and strive at making their evaluation fair trials. ICF is relevant with regard to the health condition and to the process of evaluation. Identification of grounds is a valuable instrument for controlling the quality of disability evaluation. The grounds also appear to be internationally comparable which may enhance scientific study in this area.

## Background

In their daily practice, social insurance physicians (SIPs), evaluate claims of incapacity for work. In order to conclude if a claimant is to be accepted as, and compensated for, being disabled, the SIPs always examine file information and often the claimants themselves. This process is governed by legal criteria [1-3] that are formulated in general terms that allow for tailor made decisions. These legal criteria, as far as they pertain to incapacity for work, prove to be quite comparable between countries [1,3]. All criteria contain, among other things, requirements about a claimant's health condition in relation to work, the permanence of this condition and also about claimant's responsibility to seek therapy and rehabilitation. As such, the criteria are legal representations of the concept of the handicapped role first described by Gordon [4] or the disability role as Waddell & Aylward [5] call it.

The way these legal criteria are implemented varies between countries [3,6,7]. The evaluations are carried out in Institutes of Social Insurance (ISI's) that transform the legal assignment into operational categories so as to be able to process claims on a massive scale [8,9]. This entails operationalisations of the legal criteria, conditions of work (e.g. production time) and prescriptions of work methods (e.g. report forms) [3,8,9]. In a previous study by Boer et al. the operationalisations of the legal criteria could be clustered into three categories according to their emphasis on: medical condition (disease, symptoms, impairments), functional status (limitation of activities) and/or required rehabilitative efforts [3]. In practice, operationalisations are frequently combined. In the fifteen countries under study in 2003 Boer et.al. found four different combinations of operationalisations that determine what the SIPs in different countries have to assess: medical only (e.g. Hungary), medical and functional (e.g. Belgium and Slovenia), medical and rehabilitative (e.g. France and Norway), and a combination of all three (e.g. the Netherlands). In many countries assessment of incapacity for work in social insurance is controversial in several aspects: criteria are legal and formulated in general terms offering a large decision latitude [10,11]; the assessments are carried out in complex organisational settings [12]; the basic concepts of what constitutes disability vary [13]; and the personal encounter between claimant and assessor makes up for conflicts [14-17].

In this paper we focus on the assessments and their output: the report with arguments and a conclusion. The SIPs who perform the assessments, translate individual claimant situations according to formal ISI requirements and produce reports for the ISI administration. In these reports, information is presented with a conclusion, supported by arguments. The relationship between information, arguments, and conclusions is not univocal. Identical information can lead to different arguments and conclusions in different cases. Toulmin [18] showed that arguments do not simply emerge from information, but rather stand on the application of more general grounds. With advancing age for example, we can expect physical capacity to decrease (a general ground), and so 'advanced age' can provide an argument in support of incapacity for work that is physically demanding. Advancing age can also be seen as a normal human development, not being a matter of disease (a general ground). In this perspective 'age' can be excluded from the arguments in support of incapacity for work. Consequently, different conclusions may arise in identical cases due to the fact that different grounds are being referred to. If the conclusions of the SIPs are to be legitimate, they need, among others, to refer to grounds that are recognized by all concerned.

Studies in the Netherlands [19,20] suggest that these grounds can be of different nature: legal (representing the legal criteria), scientific (representing socio-medical evidence), or social (representing social norms as to how to deal with disabled people). Meershoek ea [10] found that Dutch SIPs decisions are more social normative than scientific and that this normative dimension needs to be transparent if the quality of the assessments is to be guaranteed. This normative aspect is a problem for the SIPs as it is the base of conflicts [21]

Good understanding of argumentation and its grounds can help in developing instruments for assessment, and so help to improve quality of practice. This argumentation oriented approach would be more effective if it were not country specific. We know from earlier research [3] hat countries use different operationalisations of the legal criteria. For this reason, we studied the grounds used by SIPs in Belgium, Norway, the Netherlands and Slovenia.

Exploring the grounds in argumentation is relevant to the profession of insurance medicine itself, given the tendency towards internationalization [6], and the priority of claimants' legal security [20,22-24]. In order to study the arguments and grounds, we can analyse case descriptions. As the grounds are probably implicit, we can use group discussions to make them explicit. Focus group interviews are useful in qualitative research to find common opinions, as they show knowledge and ideas in a context, rather than as individual opinions [25,26]. In order to establish valid grounds, we need to include SIPs who perform different tasks within the schemes in the countries involved. As the focus group discussions may lead to an agreement on grounds that depend on the group characteristics we need to validate the results with SIPs other than those who participated in the focus group.

### Aim

The aim of the study was to determine which grounds are used by SIPs in different countries to support decisions about incapacity for work and to determine if the identification of these grounds can help improve the quality of assessments in social insurance practice.

## Method

### Subjects

A case of a claim for a disability pension was presented to four focus groups of SIPs working in schemes for long-term incapacity for work in Belgium, the Netherlands, Norway, and Slovenia. The case was selected for presenting a pathology and claimant profile that is met everywhere, and for not being extremely clear and so making any discussion futile. The countries were selected for representing different operationalisations of the legal criteria. The SIPs were selected for their pronounced interest in this project and for their representing different roles or tasks if applicable. To encourage discourse, we aimed at bringing together groups of eight SIPs. We succeeded in bringing together seven SIPs in Belgium, eight in the Netherlands, four in Norway, and eight in Slovenia. Table 1 presents some relevant characteristics of disability pension schemes and of participating SIPs in these countries at the time of our study.

**Table 1:** Characteristics of disability pension schemes and participants in focus groups in participating countries

Country	Name of scheme	Operationalisation of disability	Time before assessment	Partial incapacity possible	Assessment Vis à vis or file or both	SIP's concerned (nr included in focus groups)
Belgium	Invalidity Pension	Medical, rehabilitational	52 weeks	No	Both	Primary SIP (1), primary ánd secondary SIP (6)
Netherlands	WAO (Act on insurance of incapacity for work)	Medical, functional, rehabilitational	52 weeks	Yes (7 degrees)	Both	Primary SIP (4), secondary SIP (2), appeal SIP (2)
Norway	Disability Benefit scheme	Medical, rehabilitational	Not fixed	Yes (over 50%)	File	Primary SIP (3), clinical consultant (1).
Slovenia	Act on Pension and Disability Insurance	Medical, functional	Not fixed	Yes (3 degrees)	Both	Members of primary team (4), members of appeal team (3), external consultant (1)

### The case

The participants received a five-page report about a 47-year-old worker in the construction industry, based on a real case of a claim of permanent disability after two years of sick leave. This man had been treated with a lumbar laminectomy and a nerve block. He had osteoarthritis in the right knee. He complained about constant pain and bad sleep. He used medication for relief of pain and for sleeping. He was obese and moved with difficulty. He was divorced, living alone. He had tried to work as a cab driver but sitting proved to be too heavy. We selected a Dutch case as we knew from earlier research that these had the most elaborate reports. The case was translated into Norwegian and Slovenian. Where necessary, the case was adapted with regard to forms, included organizations, and time perspective to national standards in each country by the researcher from that country. The report consisted of various records: first a record from the occupational health physician on the work and sick leave of the claimant and the efforts to resume work. In this résumé, the medical history, including treatment, was described. Next, the record of the SIP was included, based on an interview with the claimant, a medical examination, and information from the physicians providing medical treatment. The items that were described in the report of the SIP matched the items that SIPs in the Netherlands need to be informed about in order to be able to take a decision on incapacity for work [20]. The items were: the opinion of the claimant on his actual and expected (in) capacities, actual complaints, medical history and general health status, life history, previous work situation, actual private situation, and social situation. The original conclusion was omitted from the case description.

### The procedure

We used a stepwise semi-structured approach in which the SIPs initially received the assignment to supplement the case with the information they would normally have in such circumstances, in such a manner that they believed it to be a regular rather than an unusual case. We took note of any proposed alterations, in order to safeguard the medical content of the case. Thereafter, the SIPs were asked to express their opinions before the group session, in terms they would normally use. We noted the conclusions and arguments in this phase as 'primary conclusions'.

Second, the participants were invited to a focus group session. The sessions took about two hours, and were chaired by the leading researcher (WB) and one other researcher. In the sessions, the participants were asked to agree on a typical judgement about the claimant's incapacity for work in line with the legal framework of their own countries. We scored these judgements as 'secondary conclusions'. Participants were free to modify case details as they considered necessary, in order to be able to reach a consensus. We noted these proposed alterations in order to safeguard the medical content of the case.

Third, having reached agreement, they were asked to name arguments that they felt supported or refuted their conclusions. These were listed and agreed upon as being acceptable arguments.

Fourth, during the focus group discussion SIPs were asked to determine the grounds on which these arguments were found to be valid. The grounds were registered and participants were asked to discuss if these grounds were valid in their normal practice of disability evaluation.

### Validation

In a group discussion, the researchers clustered the arguments and grounds produced in the focus groups into four aspects of the assessments. Next, the researchers discussed the grounds in order to identify universal phrasing. Grounds of medical evidence included in focus group arguments were recorded separately.

The grounds as redefined by the researchers were incorporated in a questionnaire that was sent to respondents in each participating country, excluding the participants of the focus groups. We aimed at at least 10 respondents in each country, using pooling that seemed most effective in each country. 20 Belgian SIPs were selected by regional staff SIPs covering the Flemish region. 20 Norwegian SIPs were randomly selected from an existing list of active SIPs in Norway. In the Netherlands SIPs were one by one randomly selected from a list of SIPs that were active in projects of professionalisation. The recruitment went on until 10 respondents had accepted to participate. In Slovenia 10 SIPs were randomly selected from the group of SIPs who worked full time as such; they all responded. Respondents were asked to indicate if the grounds as redefined by the researchers were basically always applicable in the assessment of incapacity for work in their countries.

The agreement with stated grounds by country and total of SIPs was calculated. Differences in agreement were analysed using Pearson's Chi-square test.

## Results

### The case was recognised as realistic

In all four focus groups, the case was found to be realistic. There were comments from Norway and Belgium about opportunities for rehabilitation and modifications to rehabilitation, and clarifications in the Norwegian group about the region that the claimant lived in. These modifications did not touch on the assessment of the incapacity itself.

### Agreement on the degree of disability

Before the focus group sessions, participants were asked to state their opinion of the claimant's disability. In Belgium, seven found him partially incapacitated, and two fully incapacitated. In the Netherlands, seven SIPs found the claimant to be partially incapacitated, and one found him fully incapacitated. In Norway, one found the claimant permanently fully incapacitated, and three found him to be fully but temporarily incapacitated. In Slovenia, all eight participants found the claimant to be partially incapacitated.

During the sessions, the SIPs were asked to agree on a conclusion for the claimant's disability. After discussion, a common conclusion was reached in all sessions. The Belgian group found the claimant partially incapacitated, capable of performing light unqualified work, and further rehabilitation was suggested. The Dutch group found him partially incapacitated, with restrictions in working in situations of personal risk or of risk to others, working with vibration, doing heavy work, making extreme back movements, and in working in static positions. With these restrictions, the claimant was thought capable of performing full-time work. The Norwegian group found him fully incapacitated for the moment and further rehabilitation was suggested. The Slovenian group found him partially incapacitated, fit for light, quiet work in flexible positions, but limited in walking, crouching, or heavy lifting.

### Arguments and grounds

We had asked SIPs to prepare their reasoned arguments before the group sessions. During the sessions, arguments were listed. It was discussed if all arguments were legitimate. Arguments that were proposed by individual SIPs, but rejected by the groups during discussions as not being legitimate were:

• Refusing to provide incapacity benefit may make him more ill (No)

• The regional labour market is unfavourable (No)

• With a pain syndrome he should be active (Si)

• Possibly this man wants retirement with a disability pension (Si)

• It may be a matter of age (Si)

All other arguments were noted for each country and categorized as being in favour of either (permanent) incapacity or capacity. Thereafter, all arguments were discussed in the focus groups with regard to the grounds they referred to.

In Table 2, all grounds are presented together with examples of corresponding arguments. All arguments and grounds are presented in additional file 1.

**Table 2:** Aspects of disability, grounds, and examples of arguments by country.

Grounds	Arguments	Country
*Aspect 1. Grounds on claimant's health condition*		

It is possible that a health condition is so severe that any form of work is excluded	Clinical and functional impairments are not too severe to prevent him from doing any kind of work	Be, Si
It is possible that a health condition is severe to an extent that it precludes from some work but not all work	His level of functioning is low, too reduced for partial disability.	No
Disability represents a restriction of functional capacities	Several work activities (heavy lifting and carrying etc) are well known risk factors for low back pain and should be avoided	
Capacity for work represents the ability to perform jobs	An unqualified worker can be referred to many jobs, including light work	Be
Advanced age can be a reason to accept restrictions in activities.	His age is not a big problem	Be

*Aspect 2. Grounds on a proper process of evaluation*		

Findings have to be plausible	There is pathologic evidence of damage of back and knee	Be, Nl, Si, No
Findings have to be consistent	His reduced functioning remains not fully explained with the medical findings	No
Restriction of abilities must not be explained by other factors, notably lack of motivation or opportunity to function	He might comply better with medical advice, is too fat, has a pessimistic view on his future and is inactive	Nl
In order to determine a claimant's abilities his personal experience is a source	His medication causes a lack of alertness. He notices this effect himself.	Nl
In order to determine a claimant's abilities the medical diagnosis is a source	He has pathological degeneration of his back and knees. This risks further damage. Several work activities (heavy lifting and carrying etc) are well known risk factors and should be avoided	Nl, Si, Be,
In order to determine a claimant's abilities the medication is a source	His medication causes a lack of alertness. He notices this effect himself	Nl, Si,

*Aspect 3. Grounds on treatment, rehabilitation, and time perspective*		

Disability can be accepted as permanent when all treatment options have been tried	He has had all treatment necessary	No

*Aspect 4. Grounds on efforts to recover and resume work*		

If possibilities for treatment, rehab and/or work resumption exist the claimant is requested to try these	He might comply better with medical advice, is obese, has a pessimistic view on his future and is inactive.	Nl

*Grounds of medical evidence*		

Tramadol (an opiate) can cause a lack of alertness	His medication causes a lack of alertness. He notices this effect himself	Be, Nl, Si
Heavy lifting, carrying and the like are well known risk factors for low back pain and should be avoided in the work of people who suffer from low back pain	He has pathological degeneration of his back and knees. This risks further damage. Several work activities (heavy lifting and carrying etc) are well known risk factors and should be avoided	Be, Nl, Si
Chronic low back pain is tiring and may lead to restriction of energetic activities	This kind of chronic pain is tiring and leads to restrictions in energetic activities	Be, Nl, Si, No
Pathologic damage of back and knee make complaints of back and knee plausible	There is pathologic evidence of damage of back and knee	Be, Nl, Si, No

Some arguments fit with more than one ground. The grounds are clustered around the aspect of disability evaluation that they relate to. We found four aspects, and paid separate attention to the use of medical evidence:

• the health condition of the claimant (5 grounds, 16 arguments)

• the process of evaluation, a fair trial (6 grounds, 21 arguments)

• the time perspective of recovery, treatment and rehabilitation (1 ground, 5 arguments)

• the efforts of the claimant to recover and resume work (1 ground, 5 arguments)

• medical evidence (4 grounds, 8 arguments)

### Validation

Questionnaires were returned by 14 SIPs from Belgium, 13 from Norway, and ten from both Slovenia and the Netherlands, resulting in an overall response rate of 78%. Six questions were unanswered (five from Slovenia and one from the Netherlands), out of an expected total of 799 (17 × 47). Results are given in table 3.

**Table 3:** Agreement with stated grounds, by country and total of social insurance physicians.

	**Country**				**Total%**
	
**Grounds**	Norw N = 13	Belg N = 14	Neth N = 10	Slov N = 10	
1 A condition of damaged health can be so severe that any form of work is impossible	12	14	10	10	97,9%
2 A condition of damaged health can be severe to an extent that it precludes from some work but not all work	13	14	10	10	100%
3 Disability is characterised by a restriction of functional capacities	13	12	9	9	91,5%
4 Capacity for work represents the ability to perform jobs	12	12	9	10	91,5%
5 Advanced age is no reason for disability in itself, but can be a reason to accept restrictions in activities	11	14	7	8	85,1%
6 Findings (complaints, symptoms etc) have to be plausible in order to be taken into account in the conclusion	12	10*	10	9	87,2%
7 Findings have to be consistent in order to be taken into account in the conclusion	9	10	10	10	83,0%
8 If restrictions of functional capacities are to be taken into account, they must not solely be explained by other than medical factors, notably lack of motivation or opportunity to function on the labour market	13	12*	10	10	95,7%
9 In order to determine a claimant's functional capacities his personal experience is a source of information	12	12	10	3***	80,4%
10 In order to determine a claimant's functional capacities the medical diagnosis is a source of information	10	14*	9	5**	80,9%
11 In order to determine a claimant's functional capacities the medication is a source of information	8	12	10	7	78,7%
12 Disability can be accepted as permanent when all treatment options have been tried	9	5**	6	10**	63,8%
13 The claimant has the duty to try possibilities for treatment, rehabilitation and/or work resumption when these exist	12	11	9	7	83,0%
14 Tramadol (an opiate) can cause a lack of alertness	10	14*	8	7	83,0%
15 Heavy lifting, carrying and the like are well known risk factors for low back pain and should be avoided in the work of people who suffer from low back pain	8	12	4*	9	70,2%
16 Chronic low back pain is tiring and may lead to restriction of energetic activities	10	10	4*	9	70,2%
17 Pathologic damage of back and knee make complaints of back and knee plausible	8	12	8	8	76,6%

Total grounds 1–5 (Health condition of claimant)	61	66	45	47	93,2%
Total grounds 6–11 (Process of evaluation)	64	70	59	44	84,0%
Ground 12 (Time perspective)	9	5	6	10	63,8%
Ground 13 (Obligation of claimant)	12	11	9	7	83,0%
Ground 14–17 (Medical evidence)	36	48	24	33	75,0%
Agreement in total with all grounds	82,4%	84,0%	84,1%	82,9%	83,4%

Differences in agreement are tested with Pearson Chi-square test. The contrast is subgroup vs. all other cases.

*: p < 0,05, **: p < 0,01, ***: p < 0,001 for percentages significantly higher or lower than in the entire group.

The total agreement over all items and all countries was 83.4%. The variation in total agreement between countries was very small, from 82.4% to 84.1%.

The highest agreement was reached with grounds on the health condition of the claimant, grounds 1–5 (93%). For grounds related to the proper process of evaluation, agreement was lower (84%). The lowest agreement was found for the single ground on permanence of disability (65%*). For compliance and for grounds related to medical evidence, agreement reached 83% and 75%* respectively.

When examining the agreement with individual grounds, larger differences were found, ranging from 100% (ground 2 in all countries) to 30% (ground 9 in Slovenia).

Grounds that were the least agreed upon (below 80% agreement) were grounds 11, 12, 15, 16 and 17. The most controversial grounds (between 33% and 50%) within countries are 12 in Belgium; 12, 15 and 16 in the Netherlands; 11, 15 and 17 in Norway and 9 and 10 in Slovenia.

## Discussion

### Main findings

In a series of sessions with focus groups in different countries, we studied the reasoning of SIPs in disability evaluation in public schemes for long-term incapacity for work by making arguments and grounds explicit in the case of a construction worker with low back pain. A typical case could be constructed and SIPs in three different countries were able to reach the same agreement on the conclusion of incapacity for work. In Norway, agreement was also reached, but with a different conclusion.

The arguments and grounds that were used in the focus groups were quite comparable between the countries studied. As expected the grounds represent the legal criteria of underlying health condition, compliance with therapy and rehabilitation, and permanence. In addition, six grounds relate to ubiquitous requirements of a fair trail. In the validation study, all grounds were recognized by SIPs who had not participated in the focus groups. SIPs showed high agreement on grounds with regard to the claimant's health condition, and grounds about the claimant's duty to explore opportunities for treatment, rehabilitation, and/or work resumption. Agreement was less on the ground of accepting permanent incapacity when all options for treatment had been exhausted.

### Strengths and weaknesses

A stepwise approach was used to produce these results, asking SIPs in different countries to comment on a specific case and reflect on the grounds they used. The countries were selected on the basis of different operationalisations of the legal criteria. This is not a stable fact as policies change [6,23]. The way these SIPs were recruited may have led to a selection of SIPs with a higher professional interest than average. This was a deliberate choice in order to produce the most explicit and different grounds. The Norwegian group was smaller than planned, possibly leading to higher degree of uncertainty for the results from Norway. The case was a Dutch case about low back pain. It is possible that at least some grounds are specific to the case, especially the grounds of medical evidence. Further research is needed in order to identify other grounds in other cases.

The grounds we found were clustered by the researchers into five aspects of disability evaluation, congruent with the "handicapped role" and a fair trial. The advantage of "handicapped role" is that it also useful to describe legal criteria [1,3] and so it seems fit for categorizing grounds. This is not to say that grounds could not have been clustered along other lines, for instance legal grounds next to medical grounds. The clustering into aspects showed that aspects are not mutually exclusive. For instance, the grounds that pertain to the condition of the claimant are connected to the way this condition is examined, and thus with the aspect of a proper process of evaluation. Further research may lead to more refined categories.

In the validation study, other SIPs were asked to recognize the grounds identified in the focus groups. There was a high concordance for most aspects. Quantitative research is needed in order to establish how grounds are used in daily practice.

### Other studies

In the literature we found two other studies into grounds that SIPs use in their daily practice [19,27]. In these Dutch studies, the same procedures were followed and comparable results were found. A validation with a questionnaire was not conducted in either study. We did not follow the categorization into legal, scientific, and social grounds as used in these studies, as we think that the aspects we used here are more precise.

An important model used in disability is the International Classification of Functioning and Health [28]. Slebus et.al [29] found that ICF was only partly considered by Dutch SIPs. In our study, the ICF model could be applicable to the grounds on the health condition that has to be evaluated, but not to the grounds of fair trial, rehabilitation and compliance. A complete evaluation of work disability seems therefore to contain more elements than the ICF provides.

### Impact

Studying the reasoning of individual SIPs is possible with the method we used. Differences in their personal convictions can be identified with the analysis of grounds. If our results stand in future research, a potentially important key to understanding disability evaluation has been found: the highly individual evaluations in different national contexts appear to obey rules of a specific image of disability and of legal principles of a fair trial. Being explicit about methods of assessment and about grounds used in particular cases will make the evaluations more transparent and more receptive to quality control. For example, the use of scientific evidence becomes more transparent, which seems to be relevant if we look at the differences of opinion about the effect of Tramadol. Another example is the norm of when to decide that a disability is permanent: in this study it appears to be a norm that is open to individual interpretation.

We studied specific countries because in earlier research they were found to be different in the operationalisations of the legal criteria at the level of the Institution of Social Insurance. Our results do not support that proposition on the level of the assessments. In the assessments in our study, the participants reasoned in line with the concept of the handicapped role.

## Conclusion

The identification of grounds that SIPs use helps us to understand the practice of disability evaluation. The grounds guide the translation of information into arguments about legal incapacity for work. It is possible to make these grounds explicit, as they refer to a limited group of key elements of disability evaluation.

Further research is needed to validate and extend our findings, e.g. study on the use of grounds and differences between SIPs, and subgroups of SIPs e.g. full time working vs part time and experienced vs little experienced.

SIPs interpret disability according to a concept that meets legal criteria: the handicapped role. Added to that is the concept of a fair trial. The model of disability, as described in ICF, is applicable to describe the health condition aspect of the "handicapped role". ICF also matters with regard to the process of evaluation where consistency is concerned: the consistency is to be found within and between the categories of the ICF model.

Making the grounds in disability evaluation explicit will enhance the quality of medical reports, as the grounds used can be evaluated in individual cases. In professional practice consensus about these grounds is something to strive for. This would also contribute to the transparency and legitimacy of the disability pension schemes as these schemes are open to constant criticism about their capacity to select the right people for a pension.

## Ethics committee

This study was not submitted for ethical approval. The study includes physicians who are not asked to perform specific professional actions for this study but to state and to discuss their professional opinions in general.

## Competing interests

The authors declare they have no competing interests. The study was supported with a grant from the SIG Foundation who had no involvement in the study itself or in the decision to submit the paper for publication

## Authors' contributions

WEL de Boer designed the study, did the field work and prepared the manuscript. P Donceel, S Brage and M Rus organised and chaired the focus group sessions and led the questionnaire rounds in their respective countries. They also participated in drafting and revising the manuscript. JHBM Willems supervised the project and participated in drafting and revising the manuscript.

## Pre-publication history

The pre-publication history for this paper can be accessed here:



## Supplementary Material

Additional file 1 Appendix Click here for file
