# Supplementary material for: Medico-legal reasoning in disability assessment: A focus group and validation study
Source: BMC Public Health. 2008 Sep 25;8:335. doi: 10.1186/1471-2458-8-335 (PMC2571101; doi:10.1186/1471-2458-8-335)
Supplement: Additional file 1 — Appendix [file 1471-2458-8-335-S1.doc]

Appendix : Aspects of disability, grounds, and arguments by country.

| Grounds | | | Arguments | Country |
| --- | --- | --- | --- | --- |
| *Aspect 1. Grounds on claimant’s health condition* | | |  |  |
| 10 It is possible that a health condition is so severe that any form of work is excluded | | | 10.1 Clinical and functional impairments are not too severe to prevent him from doing any kind of work | Be, Si |
|  | | |  |  |
| 11 It is possible that a health condition is severe to an extent that it precludes from some work but not all work | | | 11.1 His level of functioning is low, too reduced for partial disability. | No |
|  | | |  |  |
| 12 Disability represents a restriction of functional capacities | | | 12.1 Medication (tramadol) causes a lack of alertness | Nl, Si |
|  | | | 12.2 Several work activities (heavy lifting and carrying etc) are well known risk factors for low back pain and should be avoided | Nl, Si |
|  | 12.3 Physical examination, complaints, and functioning are consistent with each other | | | Nl |
|  | 12.4 This kind of chronic pain is tiring and leads to restrictions in energetic activities | | | Nl |
|  | 12.5 Claimant may over esteem his capacities | | | Be |
|  | 12.6 Complaints and claimed restrictions are severe | | | Be |
|  | 12.7 There are no other explanations for his reduced functioning but disease and possibly a weak motivation | | | No |
|  | 12.8 There are no signs of a lack of control of his functioning or alertness | | | Nl |
|  | 12.9 Clinical and functional impairments are not too severe to prevent him from doing any kind of work | | | Be, Si |
|  | 12.10 His reduced functioning remains not fully explained with the medical findings | | | No |
|  | 12.11 His level of functioning is low, too reduced for partial disability | | | No |
| 13 Capacity for work represents the ability to perform jobs | | | 13.1 An unqualified worker can be referred to many jobs, including light work | Be |
|  | 13.2 He has the possibility to adapt himself to other work | | | Be |
| 14 Advanced age can be a reason to accept restrictions in activities. | | | 14.1 His age is not a big problem | Be |
|  |  | | |  |
| *Aspect 2. Grounds on a proper process of evaluation* | | |  |  |
| 20 Findings have to be plausible | | | 20.1 He seems well motivated to work and follows medical advice | Nl |
|  | 20.2 His medication causes a lack of alertness. He notices this effect himself | | | Nl |
|  | 20.3 There is pathologic evidence of damage of back and knee | | | Be, Nl, Si, No |
|  | 20.4 Several work activities (heavy lifting and carrying etc) are well known risk factors and should be avoided | | | Si, Nl |
|  | 20.5 Trial of work resumption failed | | | No |
|  |  | | |  |
| 21 Findings have to be consistent | | | 21.1 Examination is consistent with some incapacity | Be |
|  | | 21.2 Physical examination, complaints and functioning are consistent with each other | | Nl |
|  | | 21.3 His reduced functioning remains not fully explained with the medical findings | | No |
|  | | 21.4 There are inconsistencies between complaints and findings | | Nl, |
|  | | 21.5 Claims to need strong medication | | Be |
|  | | 21.6 He does not complain about fatigue with his pain | | Nl |
|  | | 21.7 He has been properly examined | | No |
|  | |  | |  |
| 22 Restriction of abilities must not be explained by other factors, notably lack of motivation or opportunity to function | | | 22.1 He might comply better with medical advice, is obese, has a pessimistic view on his future and is inactive | Nl |
|  | | 22.2 He seems well motivated to work and follows medical advice | | Nl |
|  | |  | |  |
| 23 In order to determine a claimant’s abilities his personal experience is a source | | | 23.1 His medication causes a lack of alertness. He notices this effect himself. | Nl |
|  | | 23.2 Claimant may over esteem his capacities | | Be |
|  | | 23.3 Complaints and claimed restrictions are severe | | Be |
|  | | 23.4 He claims to be unable to sustain efforts with his back | | Nl |
|  | |  | |  |
| 24 In order to determine a claimant’s abilities the medical diagnosis is a source | | | 24.1 He has pathological degeneration of his back and knees. This risks further damage. Several work activities (heavy lifting and carrying etc) are well known risk factors and should be avoided | Nl, Si, Be, |
|  | | 24.2 Many people with this condition do work | | Be |
|  | |  | |  |
| 25 In order to determine a claimant’s abilities the medication is a source | | | 24.3 His medication causes a lack of alertness. He notices this effect himself | Nl, Si, |
|  | |  | |  |
| *Aspect 3. Grounds on treatment, rehabilitation, and time perspective* | | |  |  |
| 30 Disability can be accepted as permanent when all treatment options have been tried | | | 30.1 He has had all treatment necessary | No |
|  | | | 30.2 He has had all rehabilitation possible | No |
|  | | | 30.3 Trial of work resumption failed. | No |
|  | | | 30.4 It is really time he should get back to a job | Be |
|  | | | 30.5 Treating neurologist thinks further treatment useless and restrictions severe | Nl |
| *Aspect 4. Grounds on efforts to recover and resume work* | | |  |  |
| 40 If possibilities for treatment, rehab and/ or work resumption exist the claimant is requested to try these | | | 40.1 He could use a different medication. | Nl |
|  | | | 40.2 He might comply better with medical advice, is obese, has a pessimistic view on his future and is inactive. | Nl |
|  | | | 40.3 Maybe treatment options still exist | No |
|  | | | 40.4 It is really time he should get back to a job | Be |
|  | | | 40.5 Further treatment is possible, like graded activity | Nl |
| *5 Grounds of medical evidence* | | |  |  |
|  | | |  |  |
| 51 Tramadol (an opiate) can cause a lack of alertness | | | 51.1 His medication causes a lack of alertness. He notices this effect himself | Be, Nl, Si |
| 52 Heavy lifting, carrying and the like are well known risk factors for low back pain and should be avoided in the work of people who suffer from low back pain | | | 52.1 Several work activities (heavy lifting and carrying etc) are well known risk factors for low back pain and should be avoided  52.2 He has pathological degeneration of his back and knees. This risks further damage. Several work activities (heavy lifting and carrying etc) are well known risk factors and should be avoided | Be, Nl, Si |
| 53 Chronic low back pain is tiring and may lead to restriction of energetic activities | | | 53.1 This kind of chronic pain is tiring and leads to restrictions in energetic activities | Be, Nl, Si, No |
| 54 Pathologic damage of back and knee make complaints of back and knee plausible | | | 54.1 There is pathologic evidence of damage of back and knee | Be, Nl, Si, No |
